# Next-Generation Sequencing Reveals One Novel Missense Mutation in *COL1A2* Gene in an Iranian Family with *Osteogenesis imperfecta*

**DOI:** 10.18869/acadpub.ibj.21.5.338

**Published:** 2017-09

**Authors:** Farah Talebi, Farideh Ghanbari Mardasi, Javad Mohammadi Asl, Amir Hooshang Bavarsad, Masoumeh Salehi Kambo

**Affiliations:** 1 Department of Genetic, Faculty of Science, Shahid Chamran University of Ahvaz, Ahvaz, Iran; 2 Department of Midwifery, Shoushtar University of Medical Sciences, Shoushtar, Iran; 3 Department of Medical Genetics, Faculty of Medicine, Ahvaz Jundishapur University of Medical Sciences, Ahvaz, Iran; 4 Department of Internal Medicine, Faculty of Medicine, Ahvaz Jundishapur University of Medical Sciences, Ahvaz, Iran

**Keywords:** Collagen type I, *COL1A2*, Mutation, Osteogenesis imperfecta, Next-generation sequencing

## Abstract

**Background:**

Osteogenesis imperfecta (OI) is a clinically and genetically heterogeneous disorder characterized by bone loss and bone fragility. The aim of this study was to investigate the variants of three genes involved in the pathogenesis of OI.

**Methods:**

Molecular genetic analyses were performed for *COL1A1*, *COL1A2*, and *CRTAP* genes in an Iranian family with OI. The DNA samples were analyzed by next-generation sequencing (NGS) gene panel and Sanger sequencing.

**Results:**

Five different variants were identified in *COL1A1* and *COL1A2*, including two variants in *COL1A1* and three variants in *COL1A2*. Among the five causative *COL1A1* and *COL1A2* variants, one novel variants, c.1081 G>A, was found in *COL1A2*, which was identified in two siblings.

**Conclusion:**

Our finding extends the variant spectrum of the *COL1A2* gene and has important implications for genetic counseling of families. The NGS is a powerful molecular diagnostic strategy for OI, a heterogeneous disorder.

## INTRODUCTION

Osteogenesis imperfecta (OI) is a rare heritable connective tissue disorder in human that is characterized by skeletal fractures with mild trauma, extraskeletal manifestations, and secondary deformities[1,2]. In addition to such manifestations, it may include progressive hearing loss, blue sclerae, hyperlaxity of ligaments and skin, and dentinogenesis imperfecta[1,2]. The severity of OI differs from mild (OI types I, IV, and V) and severe (OI types III, IV, VI, VII, and VIII) to perinatally lethal (OI types II, VII, and VIII). OI types I-IV are caused by mutations in either *COL1A1* or *COL1A2*[3,4]. However, OI types VII and VIII are correlated with mutations in *CRTAP* and *LEPRE1*, respectively. The molecular pathogenic determinants for OI types V and VI have not been detected[3].

The inheritance patterns of OI include autosomal recessive (OI type VII and VIII) and autosomal dominant (OI type I-V)[3]. The majority of patients with OI have an identifiable variant in either *COL1A1* or *COL1A2* genes, which encode the alpha chains of collagen type I[4]. In the present study, we applied next-generation sequencing (NGS) and Sanger sequencing to analyze the whole exome of two Iranian patients with OI. A novel variant, which was not previously associated with *COL1A2*, was identified. These results will help to provide a better diagnosis of mild OI type I when physical symptoms are mild.

## MATERIALS AND METHODS

### Patients

**Fig. 1:**
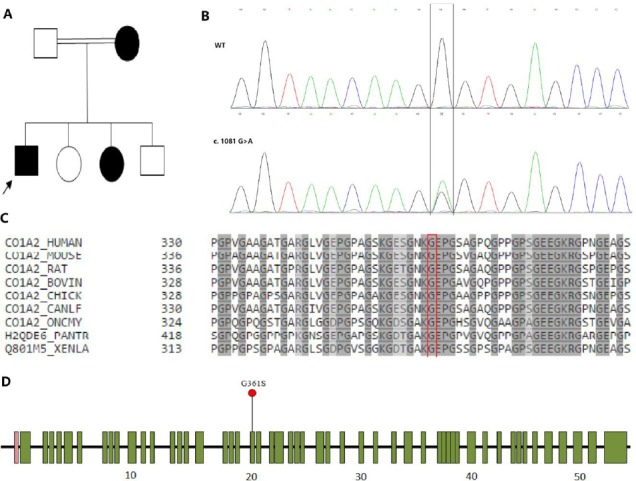
The summary of data from Osteogenesis imperfecta (OI) patients in an affected family. (A) Pedigree of family with OI shows three affected individuals in consanguineous family. The patients are denoted in black, and the proband is indicated by arrow. (B) Electropherogram analysis. The partial sequences of *COL1A2* in the patient show that heterozygous variant (c.1081 G>A) in *COL1A2* cosegregate with the phenotype. Mutated nucleotide is marked with vertical line (black). (C) Conservation analysis. Protein alignment indicates the conservation of the amino acid sequence of *COL1A2* G361S between species around the variant site. This novel variant occurs at an evolutionarily conserved residue marked with vertical line (red). (D) Schematic representation of the *COL1A2* gene showing a novel variant described in patients with OI and showing in which exon this was found.

Two siblings of a consanguineous *Iranian family* were presented with blue sclerae, dentinogenesis imperfecta, and bone pain (Fig. 1A). Each had experienced two bone fractures. The primary clinical diagnosis of OI for each individual was mainly established *through* clinical standards. Two affected family members and 200 healthy controls agreed to genetic test, and for the purpose of the study, an informed consent was obtained from legal guardians and patients. The study was approved by the Ethic Committee of Iran’s Ministry of Health and Medical Education.

### Genetic analysis

Peripheral blood samples were obtained from two patients and 200 unrelated, ethnically matched healthy controls. Genomic DNA was extracted from peripheral blood leukocytes by salting-out method according to standard instructions[5]. The quality of extracted samples was measured by using gel electrophoresis, and the quantity was identified by the use of spectrophotometric analysis. The sequencing analysis was performed with the help of a custom designed NimbleGen chip capturing of three candidate genes (*COL1A1*, *COL1A2*, and *CRTAP*) that are involved in the pathogenesis of OI, followed by NGS approach (BGI-Shenzhen, Guangdong, China).

### Bioinformatics analysis

All recognized variants were filtered out using public databases (HapMap samples and 1000 Genome Project with a minor allele frequency greater than 0.05), Ehlers-Danlos syndrome, and OI variant databases. The genomic sequence was compared with the annotated *COL1A2* gene reference sequence (NM_000089) to confirm the candidate variants. The amino acid sequences of different vertebrate species were aligned according to a HomoloGene (https://www.ncbi.nlm.nih.gov/homologene) software to investigate whether amino acid substitutions occur in the conserved regions of the proteins. The consequent phenotype of the observed novel variant was predicted using PolyPhen-2 (Polymorphism Phenotyping, v.2)[6], SIFT (Sorting Intolerant from Tolerant)[7], as well as MutationTaster predictions[8]. Once a deleterious novel variant was identified in the proband by NGS, Sanger sequencing of the mutated region was performed in two members of the family to verify its segregation with the phenotype. The novel recognized variant was subsequently investigated in 200 unrelated healthy controls with Sanger sequencing.

## RESULTS

Targeted exon capture and NGS analysis of three previously known OI genes (*COL1A1*, *COL1A2*, and *CRTAP*) were performed in an Iranian OI patient (II-1). Additionally, the mean coverage of the target region was more than 99.08%, with an average sequencing depth of more than 278× and a variant accuracy of over 99%. We identified four previously reported variants and one novel variant in *COL1A1* and *COL1A2* genes (Table 1).

**Table 1:** Summary of variants identified in the family

Gene	RefSeq	Nucleotide variant	Protein effect	Exon	Het/homo
*COL1A1*	NM-000088	c.3223 A>G	p.Thr1075Ala	48	Het
	NM-000088	c.2298 T>C	p.Thr766Thr	38	Hom
*COL1A2*	NM-000089	c.937-3 C>T	-	18	Het
	NM-000089	c.1081 G>A	p.Gly361Ser	20	Het
	NM-000089	c.1645 C>G	p.Pro549Ala	28	Het

Het, heterozygous; Hom, homozygous; RefSeq, reference sequence

After NGS, Sanger sequencing confirmed the novel heterozygous missense variant in exon 20 of the *COL1A2* gene (c.1081 G>A, Het) (Fig. 1D) in two affected members of the family (II-1 and II-3) with dominant inheritance pattern, which was absent in 200 healthy controls (Fig. 1B). The specific PCR primer encompassing *COL1A2* exon 20 was used for amplification of each DNA template[9]. Furthermore, the mother (I-2) was not accessible for genetic analysis of the identified novel variation. This variant was predicted to cause glysine to serine substitution at position 361, which is deleterious.

## DISCUSSION

OI is a disorder of bone formation that is caused by different variants of several genes. In this study, we made exact genetic diagnosis for two patients with OI due to typical clinical symptoms combined with detection of five causative variants of *COL1A1* and *COL1A2* genes via targeted NGS. We also report one novel missense variant in *COL1A2* gene in an Iranian family with OI. *COL1A2* comprises of 52 exons, which spans 36.67 kb of DNA on chromosome 7q21.3 and encodes a 1366-amino-acid protein in its longest form[10].

To date, more than 409 COL1A2 variants have been reported (http://www.hgmd.cf.ac.uk/ac/index.php), which include nonsense, missense, splicing, small deletions, small insertions, small indels, and complex variants in the 52 exons of the *COL1A2* gene. The c.1081 G>A (p.G361S) novel variant is located in α-chain of the COL1A2 protein. The glysine to serine substitution at the Gly position of a Gly-X-Y repeat results in the loss of non-polar side chains and replacement with residues containing polar sidechains. This alteration is predicted to negatively affect the function of the COL1A2 protein by disrupting its uninterrupted Gly-X-Y triplets (Fig. 1C). Most data have proposed that the substitution of glycine, as the smallest amino acid, with other amino acids such as arginine, alanine, serine, or cysteine impairs the stability and formation of the collagen triple helix, the incorporation of mature collagen into the extracellular matrix, the secretion of procollagen from the cell, and extracellular processing[11].

Phenotypic severity correlated somewhat with the type of mutation or the mutated gene. Gly missense mutations tended to lead to a more severe phenotype than other types of mutations (frameshift, nonsense, or splicing mutations). The clinical manifestations of this mutation were variable in two patients with mild OI (type I); one patient (II-1) had blue sclerae, normal

Het, heterozygous; Hom, homozygous; RefSeq, reference sequence stature, and fractures with little limb deformity, while the other (II-3) had hearing loss, fractures with no limb deformity, and blue sclerae. OI patients with the same mutation (p.Gly361Ser) showed phenotypic variability within the family. Any explanation of this phenomenon (same mutation with phenotypic variability) remains highly speculative. An epigenetic factor or a modifier gene may be involved in this variation.

In summary, the *COL1A2* c.1081 G>A variant described here in the proband and his sister, if translated, would disrupt Gly-X-Y repeat in triple helix of collagen 1 chain. This missense variant is the molecular basis of OI type I in this Iranian Family. Its identification adds further insight to *COL1A2* variants in the Iranian population with OI type I. The finding of novel variants would expand the genotypic spectrum of the *COL1A2* gene, which is valuable for better understanding of the correlation between genotype and phenotype of OI.
